# Erotomania during Typhoid Fever

**Published:** 1894-01

**Authors:** C. M. Boger

**Affiliations:** Parkersburg, W. Va.


### EROTOMANIA DURING TYPHOID FEVER.

C. M. Boger, M. D., Parkersburg, W. Va.

October 11th, at 9 A. M., was called to see Miss T., æt. fourteen, who had been under allopathic treatment for typhoid fever for three weeks. The following condition presented itself: Great restlesness, and an abject fear of being put to bed; constant endeavor to escape from the house; marching back and forth, requiring the close attention of several persons to prevent her from running against any obstacle in her path; eyes wide open with dilated pupils, and accumulation of eye gum, now and then sinks for a moment into a chair and slumbers, with open eyes and balls rolled upwards. She constantly endeavored to reach to the genitals, to expose them, or to carry the hands of her attendants to them; tries to climb up the walls, feeling all over them with trembling hands, as if in search of something she could not see; picks at the tips of her fingers, frequent urging to pass a scanty stool, which resembled cornmeal and water; urine retained, pulse 102, temperature could not be taken on account of the great restlessness. This condition persisted for five days and nights, and had resisted the administration of Morphia and Chloral by her allopathic physicians. She received at once one powder Hyoscyamus$^{200}$, dry on the tongue, to be repeated at 1 P. M. During the afternoon amelioration set in, at 11 P. M. she was asleep, and resting well, waking at 7 A. M., with no recollection of her previous condition. Her mind now was clear, and she rested well contented in bed. The improvement continued for two days, during which time she received Sac-lac. Then pulmonary symptoms called for Lachesis, of which she received two doses in five days. The last remedy cleared up all the cough symptoms, and her recovery was rapid. To-day she is the picture of health, in spite of the unfavorable prognosis of her former attendants.

